# Experience of a multi-component therapy group for patients with chronic pain

**DOI:** 10.1192/j.eurpsy.2021.1984

**Published:** 2021-08-13

**Authors:** P. Marco Coscujuela, A. Hernández Mata, A. Sotillos Gómez, C. Rodríguez Sabaté, A. Fernández Rodríguez

**Affiliations:** 1 Psychiatry, Hospital Universitario de Getafe, Getafe, Spain; 2 Psychology, Hospital Universitario de Getafe, Getafe, Spain

**Keywords:** Pain, Therapy group

## Abstract

**Introduction:**

Chronic pain has an impact that goes beyond the physical plane and, over the years, it ends up deteriorating the emotional, working and social life of people who suffer it.

**Objectives:**

Since we are working with patients who suffer a chronic pathology that cannot be cured, the objective of the group is to create a safe space in which these patients can feel understood, facilitating emotional expression and promoting an active attitude. Accepting pain and its limitations allows the person to regain their ordinary life.

**Methods:**

A multicomponent group therapy with a cognitive-behavioural orientation was carried out. The group was formed by 12 patients, all of them women with chronic pain. Eleven sessions were established on a weekly basis of one and a half hours of duration.

**Results:**

In each session a specific aspect was worked on, favouring the learning of techniques, tools and strategies of coping. A global approach was made, including behavioural, cognitive and emotional elements. At the end of the process, the patients reported benefits in their ability to manage anxiety and depression symptomatology, and they reflected a lower impact of pain in their daily life.

**Conclusions:**

Given the complexity of the symptomatology in chronic pain, it is important to approach the treatment from a multidimensional perspective that envisages every component of pain in order to being able to give a response to the physical and psychosocial impact that it implies, favouring a better confrontation and adaptation.

**Disclosure:**

No significant relationships.

